# Amino Acid Metabolism in Cancer Drug Resistance

**DOI:** 10.3390/cells11010140

**Published:** 2022-01-02

**Authors:** Hee-Chan Yoo, Jung-Min Han

**Affiliations:** 1 Yonsei Institute of Pharmaceutical Sciences, College of Pharmacy, Yonsei University, Incheon 21983, Korea; heechan@yonsei.ac.kr; 2 Department of Integrated OMICS for Biomedical Science, Yonsei University, Seoul 03722, Korea

**Keywords:** amino acids, drug resistance, cancer, immune response

## Abstract

Despite the numerous investigations on resistance mechanisms, drug resistance in cancer therapies still limits favorable outcomes in cancer patients. The complexities of the inherent characteristics of tumors, such as tumor heterogeneity and the complicated interaction within the tumor microenvironment, still hinder efforts to overcome drug resistance in cancer cells, requiring innovative approaches. In this review, we describe recent studies offering evidence for the essential roles of amino acid metabolism in driving drug resistance in cancer cells. Amino acids support cancer cells in counteracting therapies by maintaining redox homeostasis, sustaining biosynthetic processes, regulating epigenetic modification, and providing metabolic intermediates for energy generation. In addition, amino acid metabolism impacts anticancer immune responses, creating an immunosuppressive or immunoeffective microenvironment. A comprehensive understanding of amino acid metabolism as it relates to therapeutic resistance mechanisms will improve anticancer therapeutic strategies.

## 1. Introduction

Although many advances in cancer treatments have been made, the occurrence of drug resistance in cancer cells remains a challenge. Chemotherapies, targeted therapies, and immunotherapies have been effectively used as tumor treatments, whereas the emergence of drug-resistant clones leads to distant metastasis and repopulation of cancer cells, restricting clinical outcomes. Many underlying mechanisms of drug resistance have been proposed, as each tumor has the inherent characteristics of the tumor microenvironment (TME), the presence of cancer stem cells (CSCs), and heterogeneity in genetic and epigenetic signatures [1,2]. Thus, preventing drug resistance remains the most urgent unmet clinical need in cancer drug treatment.

Although metabolic alterations must occur to meet the diverse metabolic needs required for adaptation to anticancer drugs and cancer cell proliferation, metabolic reprogramming in response to anticancer drug therapies has been considered a bystander effect of biological processes induced by drugs rather than a cause of drug resistance. However, several recent studies have demonstrated that drug-specific therapeutic pressure leads to metabolic reprogramming, driving drug resistance in cancer cells [3,4]. Amino acid metabolism has been recognized as the key determinant of drug resistance in tumors, satisfying the cellular demand for maintaining redox homeostasis, energy generation, and biomass production [5,6]. In addition, intercellular or subcellular transportation of amino acids and altered metabolism induced by overexpression of amino acid transporters support cancer cell metabolism overcoming drug-induced stress [7,8]. Growing evidence indicates that suppressing or enhancing amino acid metabolism and depletion or supplementation of amino acid availability is effective in abolishing drug resistance in cancer cells [9,10].

In this review, we introduce the amino acid-driven drug resistance mechanism in tumors and highlight amino acid-dependent vulnerabilities in cancer cells that can be leveraged to improve anticancer drug therapies. We also discuss the specific roles of amino acids—in particular, immune responses to anticancer immunotherapies. We aim to describe the mechanisms underlying cancer drug resistance with respect to amino acid metabolism and anticipate future directions that can be exploited to improve drug therapy in cancer patients.

## 2. Glutamine

**Figure 1:**
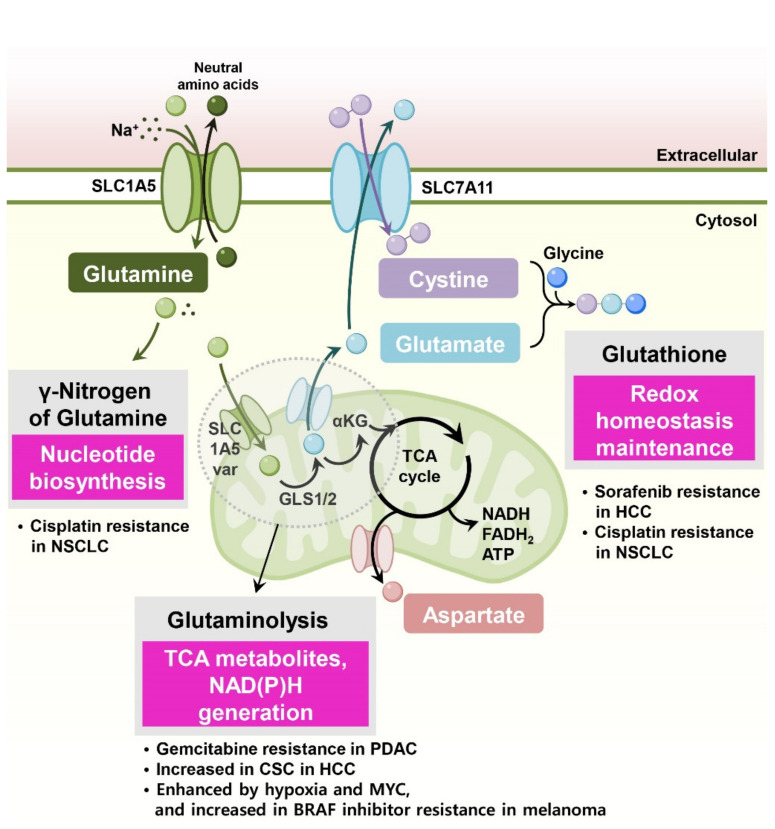
Glutamine metabolism is involved in drug resistance in cancer cells. Glutamine enters the cytosol through several plasma membrane glutamine transporters, such as SLC1A5, and is then used for nucleotide biosynthesis. For glutaminolysis, glutamine is transported into the mitochondria via the SLC1A5 variant and is subsequently catalyzed to glutamate by mitochondrial glutaminase (GLS1/2). Next, GLUD1 and several mitochondrial aminotransferases convert glutamate to α-ketoglutarate, supporting the TCA cycle reaction. Glutamine-derived TCA cycle metabolites and the generation of NADH and FADH_2_ are responsible for oxidative phosphorylation in cancer cells. Glutamine-derived glutamate serves as fuel for glutathione and is indirectly responsible for cystine uptake via the SLC7A11 transporter, which takes up cystine and simultaneously exports glutamate.

Glutamine is the most studied amino acid involved in drug resistance in cancer cells. Glutamine has a pleiotropic role in cell biology, and its dependency in several cancer types is well known. Furthermore, pharmacological intervention or dietary modulation of glutamine metabolism is considered a promising therapeutic approach. Figure 1 describes several functional mechanisms of glutamine-induced drug resistance in cancer cells.

### 2.1. Nucleotide Biosynthesis

Cytosolic glutamine supports nucleotide biosynthesis. In cisplatin-resistant human non-small-cell lung cancer (NSCLC) and ovarian cancer cell lines, glutamine is primarily required for nucleotide biosynthesis [11]. Interestingly, inhibition of mitochondrial glutaminase using BPTES or C968 did not reduce the survival of cisplatin-resistant cancer cells with glutamine dependency, suggesting that glutamine utilization for nucleotide biosynthesis in the cytosol is more important for anticancer drug resistance in these cells than fueling mitochondrial TCA cycle reactions.

### 2.2. Redox Balance

In cancer cells, glutamine supports cellular redox homeostasis by supplying fuels for glutathione synthesis and providing reducing power in the form of NADPH for managing drug-induced reactive oxygen species (ROS). In this context, cancer cells use glutamine-derived antioxidants for protecting oxidative stress prompted by therapies, resulting in drug resistance.

In tyrosine kinase inhibitor sorafenib-resistant hepatocellular carcinoma (HCC) cells, enhanced glutamine metabolism supports the survival of resistant cancer cells via the NADPH-dependent glutathione redox system [12]. HCC cells also display higher reductive glutamine metabolism, and suppressing glutamine metabolism sensitizes sorafenib-resistant HCC cells to sorafenib [12].

Cisplatin-resistant NSCLC cells potentiated glutamine-induced glutathione generation [13]. Cisplatin-resistant lung cancer cells were no longer addicted to glucose but rather relied on oxidative metabolism via glutaminolysis. Glutamine is catalyzed to glutamate for glutathione synthesis, and glutamate is directly responsible for cystine uptake via the SLC7A11 transporter. Thus, glutamine counteracts cisplatin-induced oxidative stress via glutathione production, and cisplatin-resistant cells are more susceptible to glutamine deprivation or SLC7A11 inhibition [13].

In head and neck squamous cell carcinoma (HNSCC) cells, SLC7A11 expression contributes to resistance to oxidative stress, and high expression of SLC7A11 and SLC1A5 is correlated with the dedifferentiation status of cancer cells [14]. Furthermore, SLC1A5-mediated glutamine uptake and glutamate dehydrogenase (GLUD)-mediated α-ketoglutarate production control ROS generation and determine the sensitivity of the SLC7A11 inhibitor sulfasalazine [14].

Under hypoxic conditions, cancer cells reprogram their glutamine metabolism, enhancing reductive carboxylation for fatty acid synthesis [15] and the generation of NADPH and glutathione [16]. The mitochondrial glutamine transporter SLC1A5_var induced by HIF2α confers gemcitabine resistance in pancreatic cancer cells by suppressing ROS production through glutamine-derived glutathione synthesis [17].

Cancer stem cells (CSCs) are considered to be largely responsible for drug resistance, metastasis, and tumor relapse [18]. In liver cancer, mitochondrial glutaminase (GLS1) is highly expressed, and its expression is associated with a stemness phenotype and aggressive clinicopathological features [19]. Active GLS1 elevates the levels of glutamate–cysteine ligase catalytic subunit (GCLC), the first rate-limiting enzyme in glutathione synthesis, and maintains stemness in HCC through redox signaling. Glutamine deprivation or GLS inhibitor treatment increased intracellular ROS levels and thus decreased in vivo tumorigenicity [19]. Consistently, in lung cancer cells, glutamine deprivation or pharmacological depletion of glutamine using L-asparaginase decreased the proportion of CSC-like cancer cells in vitro [20]. These treatments led to reduced cellular glutathione and increased ROS accumulation in lung cancer cells, suppressing tumor formation in in vivo xenograft models [20].

### 2.3. Oxidative Metabolism

Glutamine-derived α-ketoglutarate enters the TCA cycle, and its subsequent oxidization generates two molecules of NADH and one molecule of FADH_2_. Electron transport chain complexes use these molecules to create the electrochemical gradient necessary for ATP synthesis via oxidative phosphorylation. Additionally, glutamine-derived TCA cycle metabolites participate in the generation of nonessential amino acids, fatty acids, and nucleotides. In this context, cancer cells use glutaminolysis-induced oxidative phosphorylation and glutaminolysis-derived metabolites to mediate drug resistance.

In BRAF-mutant (V600E) melanoma cells, the Myc transcription factor and Myc-activated glutamine metabolism are essential for resistance to BRAF inhibitors. In particular, Myc-enhanced glutaminolysis supports fatty acid and pyrimidine synthesis in the resistance to BRAF inhibitors [21]. Similarly, BRAF inhibitor PLX4720-resistant melanoma cells also exhibit increased glutaminolysis, mitochondrial biogenesis, and oxidative metabolism [22]. Furthermore, suppression of glutaminolysis with glutaminase inhibitor BPTES significantly reduced respiration in BRAF inhibitor vemurafenib-resistant cells and blocked the growth of vemurafenib-resistant tumors [23].

In contrast to glutamine-induced BRAF inhibitor resistance, other studies have proposed a model in which low levels of glutamine in tumor core regions induce resistance to BRAF inhibitors [24,25]. These studies indicate that the core region of solid tumors often displays glutamine deficiency, decreased levels of α-ketoglutarate, and cancer cell dedifferentiation. They also demonstrated that dietary glutamine supplementation sensitized melanoma cells to the BRAF inhibitor PLX4032 by downregulating mitogen-activated protein kinase (MAPK) and other oncogenic pathways [25]. Contradictory results of BRAF inhibitor resistance associated with glutamine metabolism should be carefully evaluated with respect to the experimental conditions, such as whether they are in vitro or in vivo, and the relative definition of drug resistance used in each study.

Tumor hypoxia reduces the response to anticancer therapies in many cancer types [26]. The hypoxia-induced transcription factor switch from ERα to HIF1α leads to sustained glutamine metabolism via upregulation of the glutamine transporter SLC38A2 under hypoxic conditions [27]. A combination of SLC38A2 depletion using the ER antagonist fulvestrant effectively reduced mitochondrial respiration. In addition, SLC38A2 is induced during the process of gaining tamoxifen resistance, and SLC38A2 overexpression induces strong resistance to antiestrogen therapy in vivo, suggesting glutamine metabolism-driven antiestrogen resistance [27].

## 3. Asparagine

**Figure 2:**
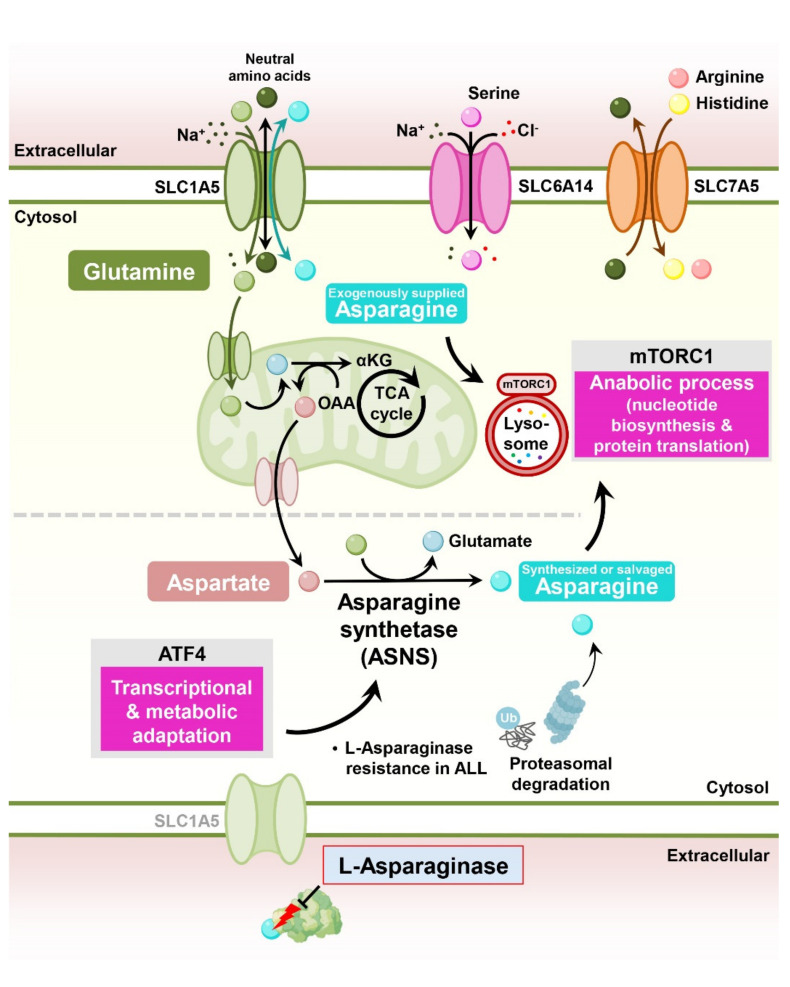
Asparagine metabolism in L-asparaginase resistance in cancer cells. Asparagine and glutamine enter the cytosol through SLC1A5. Intracellular asparagine participates in the uptake of several amino acids, especially serine, arginine, and histidine; stimulates mTORC1 signaling; and suppresses stress-activated ATF4 transcriptional activity. Through glutaminolysis and subsequent transamination reactions, glutamine accelerates intracellular aspartate and asparagine synthesis. L-asparaginase treatment depletes extracellular glutamine and asparagine and suppresses the proliferation of ALL. During the development of resistance to L-asparaginase, ALL cells express asparagine synthetase (ASNS) via ATF4, and ASNS synthesizes asparagine using glutamine and aspartate. Proteasomal degradation also supports L-asparaginase resistance, supplying salvaged asparagine via proteasomal degradation.

Asparagine, a nonessential amino acid, plays an important role in cancer cell proliferation, supporting cell survival under glutamine deprivation, electron transport chain inhibition, and metastasis in solid tumors [28]. Mechanistically, asparagine functions as an exchange factor for the uptake of other essential amino acids, stimulating mTORC1 signaling and nucleotide biosynthesis [29,30]. Interestingly, the therapy that depletes circulating asparagine in the blood using L-asparaginase is a universal therapy used in pediatric acute lymphoblastic leukemia (ALL) patients [31] (Figure 2).

A key reason for resistance to L-asparaginase is the expression of asparagine synthetase, the rate-determining enzyme for the biosynthesis of asparagine in cancer cells [32]. Exogenous expression of asparagine synthetase is sufficient to induce resistance in L-asparaginase-sensitive leukemic cells [33]. Moreover, the ability of ALL cells to properly induce asparagine synthetase under L-asparaginase treatment is essential for resistance to L-asparaginase [34,35]. Interestingly, one study showed that intracellular asparagine levels can be modulated by the control of protein catabolic flux regardless of asparagine synthetase expression. Genome-wide CRISPR screening revealed that activation of Wnt signaling sensitizes L-asparaginase-resistant ALL cells to this enzyme through the inhibition of proteasomal degradation, a catabolic source of asparagine [36].

In addition to asparagine synthetase expression in cancer cells, extrinsic factors also contribute to L-asparaginase resistance. In ALL, bone marrow-derived mesenchymal cells (MSCs) highly express asparagine synthetase compared to ALL cells, which protects ALL cells from asparaginase cytotoxicity. Asparagine secretion from MSCs is directly regulated by the asparagine synthetase expression of MSCs, and depletion of asparagine synthetase in MSCs sensitizes ALL cells to L-asparaginase treatment [37].

## 4. Methionine

Methionine is an essential amino acid for protein synthesis, one-carbon metabolism, sulfur metabolism, epigenetic modification, and redox maintenance [38]. Growing evidence indicates that modulating methionine metabolism, which coordinates nucleotide and redox status in cancer and immune cells, may induce metabolic vulnerabilities in drug-resistant tumors.

### 4.1. Folate Cycle and Nucleotide Biosynthesis

**Figure 3:**
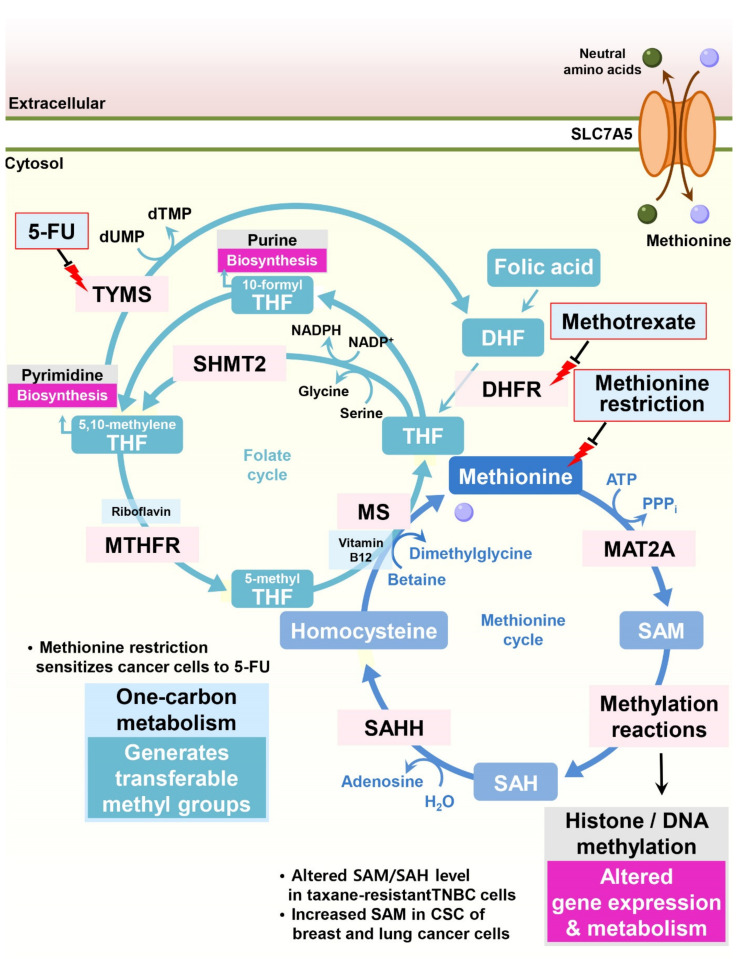
Methionine metabolism is involved in drug resistance in cancer cells. One-carbon metabolism comprises both the folate and methionine cycles, and methionine is a key component of this metabolic network. In the folate cycle, MTHFR reduces 5,10-methylene tetrahydrofolate to 5-methyl tetrahydrofolate, and then 5-methyl tetrahydrofolate transfers its methyl group to convert homocysteine to methionine by MS, initiating the methionine cycle. Methionine is catalyzed by MAT2A, producing the universal methyl donor SAM for proteins and DNA methylation. Various methyltransferases consume SAM as a source of methylation reactions, consequently generating SAH. SAHH removes the adenyl group of SAH to homocysteine. During the folate cycle, tetrahydrofolate functions as a carrier that donates one-carbon groups from serine to different molecules, such as thymidylates, purines, methionine, and SAM. To donate the one-carbon groups, tetrahydrofolate undergoes alterations of its oxidation states, such as 10-formyl tetrahydrofolate, 5,10-methylene tetrahydrofolate, and 5-methyl tetrahydrofolate. The anticancer agents 5-FU and methotrexate suppress one-carbon metabolism, inhibiting TYMS and DHFR, respectively. By impacting one-carbon metabolism, methionine restriction can sensitize resistant cancer cells to several anticancer agents. SAM: S-adenosylmethionine; SAH: S-adenosylhomocysteine; DHF: dihydrofolate; THF: tetrahydrofolate; MAT2A: methionine adenosyltransferase 2A; SAHH: S-adenosylhomocysteine hydrolase; DHFR: dihydrofolate reductase; SHMT2: serine hydroxymethyltransferase-2; MTHFR: methylenetetrahydrofolate reductase; TYMS: thymidylate synthetase; 5-FU: 5-fluorouracil.

Through the contribution of homocysteine, methionine participates in the folate cycle, which provides multiple inputs for both purine and pyrimidine biosynthesis (Figure 3). Since cancer cells display highly active nucleotide biosynthesis for proliferation and in response to methotrexate, a widely used cancer chemotherapy that inhibits the essential folate cycle enzyme dihydrofolate reductase (DHFR), modulating methionine metabolism could be a potential anticancer strategy.

Maximizing the efficacy of cancer therapy by limiting dietary methionine is an attractive treatment option with high feasibility [39]. The ability of dietary methionine restriction to modulate histone methylation status through one-carbon metabolism and related epigenetic modifications in vivo provides a mechanism for dietary methionine restriction therapy. Indeed, cellular or in vivo methionine restriction changes intracellular S-adenosyl methionine (SAM) and S-adenosyl homocysteine (SAH) levels and H3K4me3 histone methylation, resulting in altered gene expression and metabolism [40]. In addition, in patient-derived xenograft models of RAS-driven colorectal cancer, dietary methionine restriction sensitizes cancer cells to 5-fluorouracil (5-FU) [41]. Under dietary methionine restriction, cancer cells are forced to increase methionine production from homocysteine consuming intracellular 5,10-methylene-tetrahydrofolate (CH2-THF), resulting in decreased folate cycle-related metabolites and nucleotide biosynthesis [42].

### 4.2. One-Carbon Metabolism

One-carbon metabolism comprises both the folate and methionine cycles and provides transferrable methyl groups for cellular methylation reactions. Cellular DNA or histone methylation status is determined by the activity of methyltransferases and demethylases and is frequently altered in pathological states [43]. SAM derived from methionine is the dominant methyl donor for these enzymes, linking one-carbon metabolism to cellular methylation status.

In taxane-resistant triple-negative breast cancer (TNBC) cells, significant concomitant alterations in methionine and nucleotide metabolism occur. Although partial deprivation of methionine had little effect on the proliferation of parental TNBC cells, it significantly reduced the proliferation of taxane-resistant TNBC cells. Decreased incorporation of C^13^-labeled methionine into SAM and SAH was observed in taxane-resistant TNBC cells, resulting in DNA hypomethylation [44].

Since transient methionine restriction induces the differentiation of embryonic stem cells and induced pluripotent stem (iPS) cells [45], methionine restriction may reduce the stemness of CSCs, resulting in improved treatment outcomes. Indeed, methionine restriction inhibits mammosphere formation and reduces the high-CD44- and low-CD24-expressing CSC population in breast cancer cells, sensitizing CSCs to inhibition of the enzyme MAT2A, which converts methionine to SAM [46]. Additionally, methionine restriction primes TNBC tumors to respond to proapoptotic TRAIL receptor agonists by increasing the cell surface expression of TRAIL-receptor 2. Thus, methionine depletion sensitizes TNBC cells to the TRAIL receptor agonist lexatumumab and induces apoptosis of TNBC cells in vitro and in vivo [47]. Likewise, tumor-initiating cells (also called CSCs) derived from resected primary NSCLC adenocarcinoma samples and grown as non-adherent tumor spheres display increased methionine cycle metabolites, including SAM, and dependency on exogenous methionine. The small molecule MAT2A inhibitor FIDAS-5, which perturbs the methionine metabolic cycle, potently reduces intracellular levels of SAM and SAH and the tumorigenic potential of tumor-initiating cells by altering the methylation status of histones [48].

## 5. Aspartate

**Figure 4:**
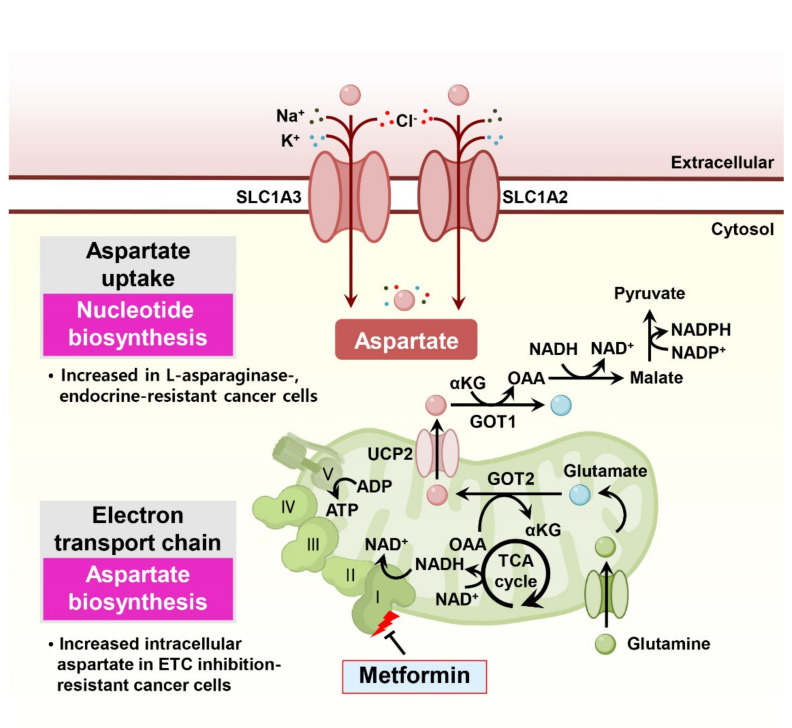
Aspartate metabolism is involved in drug resistance in cancer cells. Normal respiring cancer cells utilize glutaminolysis and oxidative phosphorylation reactions to support aspartate biosynthesis for proliferation. In particular, complex I supports cancer cell proliferation via NAD^+^ regeneration to maintain the cellular NAD^+^/NADH balance and aspartate production. Mitochondrial aspartate is transported into the cytosol through UCP2 and is used to generate NAD^+^ or NADPH. Increasing aspartate import through SLC1A3 or SLC1A2 provides advantages to cancer cells for nucleotide synthesis and the capacity of survival at low oxygen states, such as hypoxia. α-KG: α-ketoglutarate; OAA: oxaloacetate; GOT: glutamic oxaloacetic transaminase; UCP2: uncoupling protein 2; ETC: electron transport chain.

Aspartate, a nonessential amino acid that supplements TCA cycle metabolites, sustains NAD^+^/NADH homeostasis and is responsible for nucleotide biosynthesis. In particular, aspartate is essential for cell proliferation under impaired electron transport chain conditions and is associated with drug resistance in cancer cells (Figure 4).

### 5.1. Electron Transport Chain

Interestingly, in proliferating cells, the dominant function of the electron transport chain is not to generate ATP but rather to consistently provide the electron acceptor NAD^+^ for maintaining cellular redox homeostasis [49]. Thus, reduced cell proliferation in response to electron transport chain suppression through various molecules, including metformin, can be restored by supraphysiological concentrations of exogenous aspartate or pyruvate catalytically supplying NAD^+^ [50,51]. In this context, the anticancer efficacy of electron transport chain-targeting compounds, such as metformin or phenformin, could be determined by the environmental aspartate availability or the aspartate uptake capacity of cancer cells. Indeed, a previous study suggested that the anti-proliferative effect of metformin resulted from the loss of NAD^+^/NADH homeostasis and the inhibition of aspartate biosynthesis [52]. Thus, environmental aspartate availability or the activity of other pathways that affect NAD^+^ regeneration should be considered a critical determinant of the sensitivity of cancer cells to drugs targeting the electron transport chain.

Since hypoxia can limit electron transport chain function, leading to reduced electron acceptors and aspartate biosynthesis in primary tumors [53], modulating aspartate bioavailability around the tumor could be a viable therapeutic strategy. Indeed, exogenous expression of gpASNase1, which converts intracellular asparagine to aspartate, or overexpression of the aspartate transporter SLC1A3, facilitating cancer cells to take up environmental aspartate, significantly increases tumor growth in vivo [54,55].

### 5.2. Catabolic Pathway

Although L-asparaginase is an effective drug for adolescent acute lymphoblastic leukemia [31], the therapeutic efficacy of L-asparaginase in other solid tumors is not satisfactory due to intolerable toxicity in patients and L-asparaginase resistance of the tumor [56]. Interestingly, one study showed that treatment with L-asparaginase in solid tumors enhances aspartate and glutamate consumption via SLC1A3, promoting cancer cell proliferation. Consistently, treatment of L-asparaginase with the SLC1A3 pharmacological inhibitor TFB-TBOA effectively hinders cancer cell proliferation in vitro and in vivo [57].

In endocrine-resistant estrogen receptor (ER)-positive breast cancers, increased levels of intracellular aspartate and glutamate sustain the aggressive phenotype of therapeutic resistance. In endocrine-resistant breast cancer cells, levels of the neutral amino acid transporter SLC6A14 are decreased, but the expression and activity of SLC1A2, which imports the acidic amino acids aspartate and glutamate, is increased [58]. Similarly, epithelial-mesenchymal transition (EMT) of prostate cancer cells leads to metabolic reprogramming, resulting in elevated aspartate metabolism [59].

In pancreatic cancer, glutamine-mediated nicotinamide adenine dinucleotide phosphate (NADPH) production is important for balancing cellular redox homeostasis and gemcitabine resistance [17,60]. In this process, the transportation of mitochondrial aspartate derived from glutamine into the cytosol through UCP2 is essential for providing metabolic precursors for NADPH generation [61]. Indeed, UCP2 overexpression decreases mitochondrial ROS induction in response to gemcitabine and protects cancer cells from gemcitabine-induced apoptosis, suggesting the potential importance of aspartate-associated drug resistance in cancer cells [62].

Acute myeloid leukemia (AML) cells exhibit transient metabolic changes in response to chemotherapy [63]. During cytarabine- and doxorubicin-based induction chemotherapy (iCT), massive cancer cell death occurs, but immediately thereafter, persisting AML cells appear, harboring chemotherapy-induced metabolic changes that increase pyrimidine and glutathione biosynthesis. This metabolic adaptation is supported by a subpopulation of leptin receptor-positive and CXCL12-positive mesenchymal stromal cells that provide glutamine-derived aspartate through SLC1A3. Suppressing aspartate biosynthesis in bone marrow stromal cells (BMSCs) sensitizes AML cells to chemotherapy, implying that BMSC-derived aspartate induces iCT resistance in AML [63].

## 6. Branched-Chain Amino Acids, Leucine, Isoleucine, and Valine

**Figure 5:**
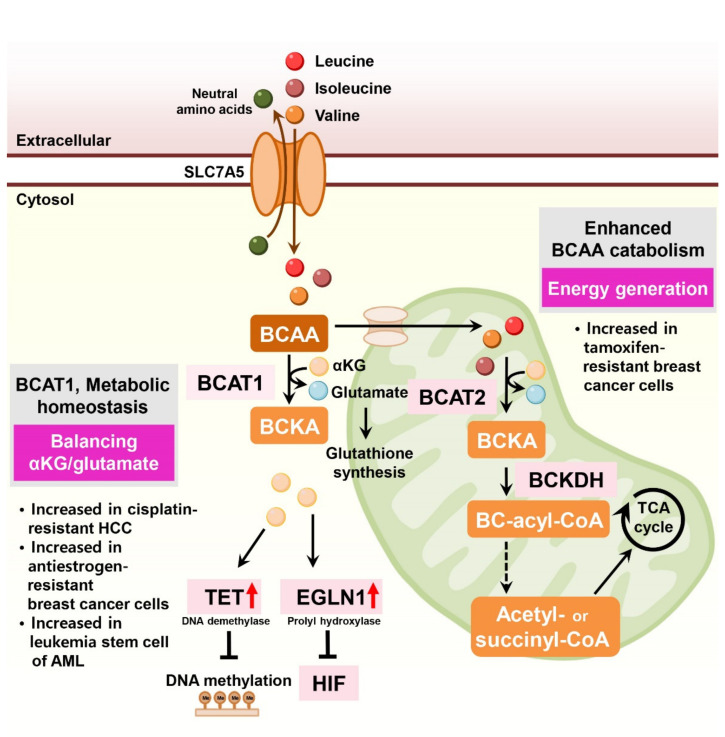
BCAA metabolism is involved in drug resistance in cancer cells. BCAAs enter the cell through SLC7A5, a key transporter that transports large neutral amino acids. In both the cytosol and mitochondria, BCAT1 and BCAT2 transfer BCAA-derived nitrogen to α-ketoglutarate to generate glutamate and BCKA. In turn, BCKA is catabolized by BCKDH to produce BC-acyl-CoA, which can be further catabolized in several steps to acetyl-CoA or succinyl-CoA. In addition to regulating BCAA and BCKA levels, BCAT is important for the homeostasis of intracellular α-ketoglutarate and glutamate levels. Overexpression of BCAT reduces the ratio of α-ketoglutarate to glutamate, resulting in DNA hypermethylation and HIFα stabilization. BCAA: branched-chain amino acid; BCKA: branched-chain keto acid; BC-acyl-CoA: branched-chain acyl-CoA; α-KG: α-ketoglutarate; BCAT: BCAA transaminases; BCKDH: branched-chain ketoacid dehydrogenase complex; TET: ten–eleven translocation; EGLN: Egl nine homolog 1; HIF: hypoxia-inducible factor.

The branched-chain amino acids (BCAAs) leucine, isoleucine, and valine are essential for cancer cell growth, activating the mechanistic target of rapamycin complex 1 (mTORC1), and supplying carbon sources for energy production [64]. Multiple studies have reported that BCAA transaminase 1 (BCAT1), the rate-limiting enzyme of BCAA catabolism, is associated with tumor aggressiveness and drug resistance in several tumor types [65,66] (Figure 5).

In liver cancer, BCAT1 expression is significantly elevated in HCC tissues compared to non-tumor tissues. Ectopic expression of BCAT1 increases tumorigenic properties and endows cisplatin resistance in HCC cells [67,68]. In human AML, the BCAA pathway is enriched, and BCAT1 expression is elevated in leukemia stem cells [69]. In these cells, BCAT1 transfers α-amino groups from BCAAs to α-ketoglutarate and maintains α-ketoglutarate homeostasis. BCAT1 depletion in leukemia cells leads to the accumulation of α-ketoglutarate, resulting in enhanced Egl nine homolog 1 (EGLN1)-mediated HIF1α degradation. Conversely, overexpression of BCAT1 in leukemia cells reduced intracellular α-ketoglutarate levels and led to DNA hypermethylation via α-ketoglutarate-dependent ten–eleven translocation (TET) DNA demethylase activity [69].

In antiestrogen-resistant breast cancer cells, BCAT1 is the most highly upregulated transcript compared to antiestrogen-sensitive breast cancer cells [70]. BCAT1 is primarily expressed in estrogen receptor-negative and human epidermal growth factor receptor 2-positive (ER-negative/HER2-positive) cancers and TNBC. BCAT1 overexpression induces antiestrogen-sensitive cells to resist antiestrogen treatments [70].

In ER-positive breast cancer cells, LLGL2 is overexpressed and sustains cell proliferation under nutritional stress conditions [71]. Mechanistically, LLGL2 controls the BCAA transporter SLC7A5 and forms a trimeric complex with SLC7A5 and a regulator of membrane fusion, YKT6, increasing leucine uptake and cell proliferation. LLGL2-dependent SLC7A5 function in nutrient stress confers resistance to tamoxifen treatment [71]. Indeed, depletion of SLC7A5 and its counterpart SLC3A2 decreased the growth of ER-positive breast cancer cells and sensitized them to tamoxifen [72]. Similarly, SLC7A5 is involved in treatment resistance and drug sensitivity in luminal-type breast cancer, contributing to energy generation via TCA cycling [73].

In lung cancer cells, sub-lethal tyrosine kinase inhibitor (TKI) treatment causes drug resistance in EGFR-mutant lung cancer cells via H3K9me2-mediated reprogramming of BCAA metabolism. This metabolic reprogramming upregulates BCAT1 and attenuates ROS accumulation [74].

## 7. Serine

**Figure 6:**
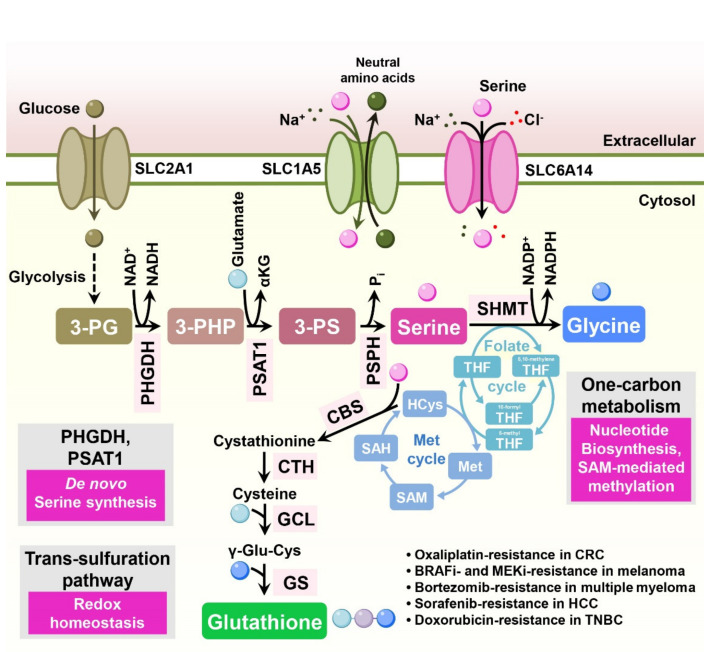
Serine metabolism is involved in drug resistance in cancer cells. Serine is synthesized de novo through the serine synthesis pathway. The glycolytic intermediate 3-PG is converted to 3-PHP by PHGDH. PSAT1 then catalyzes 3-PHP to 3-PS, which is, in turn, dephosphorylated to serine by PSPH. Serine also directly enters the cytosol through plasma membrane serine transporters, such as SLC1A4 and SLC6A14. Serine is converted to glycine by the reaction of SHMT, donating a carbon group to tetrahydrofolate and initiating one-carbon metabolism. During the methionine cycle, which is a tightly linked folate cycle, SAH is converted into homocysteine, which contributes to the transsulfuration pathway for glutathione synthesis. 3-PG: 3-phospho-glycerate; 3-PHP: 3-phospho-hydroxypyruvate; 3-PS: 3-phospho-serine; γ-Glu-Cys: gamma-glutamylcysteine; PHGDH: phosphoglycerate dehydrogenase; PSAT1: phosphoserine aminotransferase 1; PSPH: phosphoserine phosphatase; SHMT: serine hydroxymethyltransferase; CBS: cystathionine-β-synthase; CTH: cystathionase; GCL: glutamylcysteine ligase; GS: glutathione synthase; THF: tetrahydrofolate; SAM: S-adenosyl methionine; SAH: S-adenosyl homocysteine; HCys: homocysteine.

Enhanced serine metabolism has been reported in multiple tumor types [75,76]. Serine is a precursor of the amino acids glycine and cysteine, purine nucleotides, and glutathione [77]. Furthermore, serine supports one-carbon metabolism, supplying a carbon source [78]. Several studies indicate that serine metabolism is involved in drug resistance in cancer treatment (Figure 6).

While extracellular serine is sufficient for cancer cell growth, some tumors essentially require serine biosynthesis for their biological functions [77]. Serine can be synthesized from the glycolytic intermediate 3-phosphoglycerate (3-PG) via several enzymes, such as phosphoglycerate dehydrogenase (PHGDH), phosphoserine aminotransferase 1 (PSAT1), and phosphoserine phosphatase (PSPH) (Figure 6).

In colorectal cancer cells, overexpression of PSAT1 induces enhanced tumorigenic properties compared to control cells in a xenograft mouse model and confers resistance to oxaliplatin treatment [79]. Similarly, both depletion of PSAT1 in colorectal cancer cells and removal of serine from mouse diet inhibit tumor growth and increase the antitumor efficacy of 5-FU in vivo [80]. Consistently, in esophageal squamous cell carcinoma (ESCC) tissues, expression of PSAT1 is increased compared to adjacent non-cancer tissues and is significantly associated with disease stage [81]. In BRAF inhibitor-resistant melanoma cells, expression of the serine biosynthetic enzymes PHGDH, PSAT1, and PSPH is enhanced to support folate cycle metabolism, and depletion of PHGDH sensitizes resistant cells to BRAF inhibitors [82].

In multiple myeloma, cancer cells occasionally gain bortezomib resistance, enhancing serine biosynthesis through PHGDH and ultimately leading to increased antioxidant capacity across different multiple myeloma cell lines [83]. In HCC, CRISPR screening identified PHGDH as an essential driver of sorafenib resistance. Inactivation of PHGDH decreases the production of α-ketoglutarate, serine, and NADPH, consequently elevating ROS levels and sensitizing resistant cells to sorafenib treatment. Moreover, combined PHGDH inhibition and sorafenib treatment synergistically decreased tumor growth in vivo [84].

Melanoma cells harboring NRAS mutations frequently exhibit resistance to MAPK kinase inhibitors. In human NRAS-mutant melanoma xenograft models, upregulation of serine biosynthesis and expression of PHGDH are responsible for the resistance to MAPK kinase inhibitors. Depletion of PHGDH in resistant cells together with administration of a MAPK kinase inhibitor reduced glutathione levels and cell proliferation [85].

In triple-negative breast cancer cells, knockdown of PHGDH decreased intracellular glutathione and sensitized resistant cells to doxorubicin-induced oxidative stress [86]. In EGFR mutation-positive lung adenocarcinomas, downregulation of PHGDH or treatment with a PHGDH inhibitor increased ROS stress and DNA damage, ultimately sensitizing cells to receptor tyrosine kinase inhibitors (erlotinib) [87].

The serine catabolic enzyme, mitochondrial serine hydroxymethyltransferase 2 (SHMT2), is induced when Myc-transformed cells are subjected to hypoxia. Depletion of SHMT2 in these cells decreases the cellular NADPH/NADP^+^ ratio and increases cellular ROS and hypoxia-induced cell death [88]. Hypoxia-induced expression of PHGDH and SHMT2 was also reported in glioma cell lines and breast cancer stem cells (BCSCs), and knockdown of PHGDH leads to a reduced level of NADPH, elevated ROS, and increased apoptosis under hypoxia [89,90].

In contrast to other cancers, the combination of cisplatin and PHGDH inhibitors (NCT-503 or CBR-5884) reduced cisplatin-induced DNA damage in gastric cancer cells. In this study, PHGDH inhibitors decreased H3K4me3 and subsequently enhanced chromatic compactness, resulting in relieved cisplatin-induced DNA damage [91].

## 8. Lysine

In CD110-positive TICs of colorectal cancer (CRC) cells, thrombopoietin induces the saccharopine pathway (lysine catabolic pathway) and drives liver metastasis. Mechanistically, lysine-derived acetyl-CoA is significantly increased in CD110-positive TICs, enhances acetylation of the LRP6 coreceptor of Wnt signaling, and stimulates self-renewal of TICs. Lysine-derived glutamate also maintains redox homeostasis, supports drug resistance, and facilitates liver colonization [92].

## 9. Histidine

**Figure 7:**
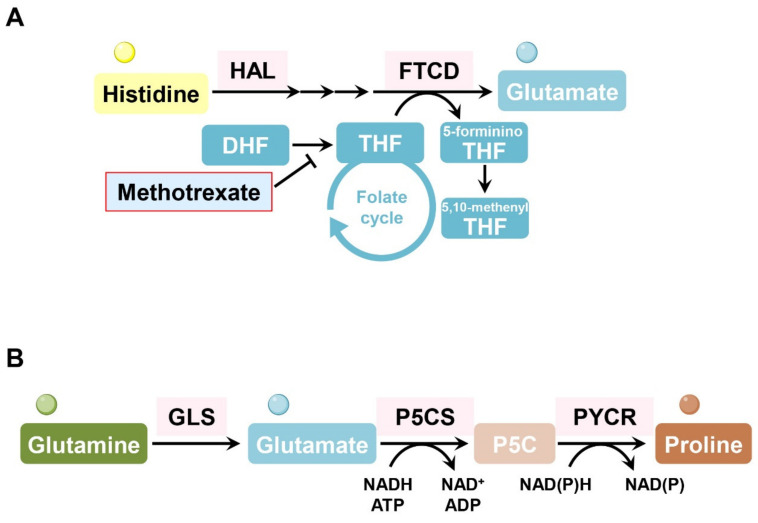
Histidine and proline metabolism is involved in drug resistance in cancer cells. (**A**) Histidine is deaminated via HAL and hydrated in its catabolic process. Its imidazole ring is cleaved to form formiminoglutamate, and then the formimino group is transferred to tetrahydrofolate, generating glutamate and formimino-tetrahydrofolate by FTCD. Consuming tetrahydrofolate through histidine catabolism depletes the cellular pool of tetrahydrofolate, which is harmful to methotrexate-treated cells. (**B**) The nonessential amino acid proline is synthesized from glutamine in the mitochondria through PYCR1 and PYCR2 or from ornithine in the cytosol through PYCR3. HAL: histidine ammonia-lyase; FTCD: formimidoyltransferase cyclodeaminase; THF: tetrahydrofolate; DHF: dihydrofolate; P5CS: pyrroline-5-carboxylate synthetase; P5C: pyrroline-5-carboxylate; PYCR: pyrroline-5-carboxylate reductase.

In several hematopoietic malignant cell types (erythroleukemia cells, Burkitt’s lymphoma cells, chronic myeloid leukemia cells), formimidoyltransferase cyclodeaminase (FTCD) and histidine ammonia lyase (HAL), which are enzymes involved in histidine catabolism consuming tetrahydrofolate, are associated with methotrexate sensitivity. Interestingly, loss of FTCD or HAL enables cancer cells to maintain cellular tetrahydrofolate levels and nucleotide synthesis, even under methotrexate treatment. Consumption of tetrahydrofolate by FTCD or HAL is particularly harmful to methotrexate-treated cells whose tetrahydrofolate levels are already low. Forced histidine catabolism through histidine supplementation combined with methotrexate significantly decreased tumor size and induced cancer cell death in vivo [93] (Figure 7A).

## 10. Proline

Treatment with L-asparaginase induces proline metabolism dependency in kidney cancer, exhibiting high levels of PYCR1, a key enzyme in proline production. Suppression of PYCR1 attenuated kidney cancer cell growth when proline was restricted [94] (Figure 7B). The hypoxic microenvironment in the TME also activates proline metabolism, resulting in the accumulation of glutamine-derived hydroxyproline, which promotes HCC progression and sorafenib resistance by stabilizing the HIF1α protein [95].

## 11. Others

### 11.1. Glutathione (Glu-Cys-Gly)

**Figure 8:**
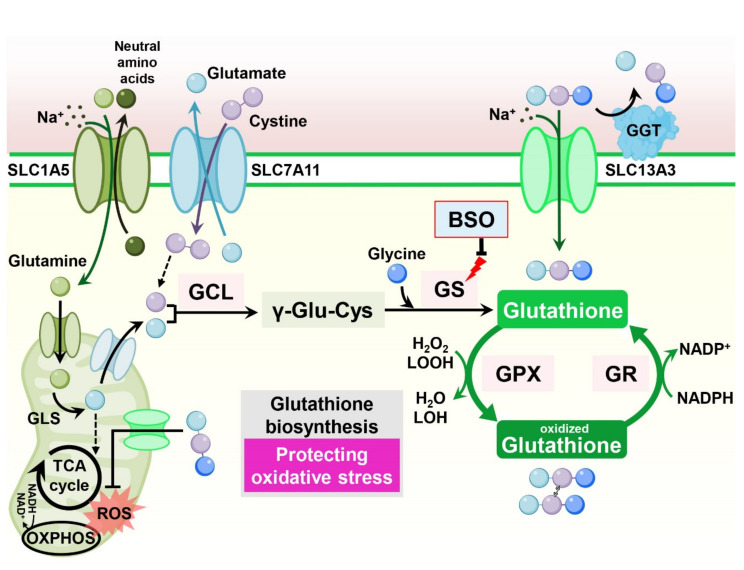
Glutathione metabolism is involved in drug resistance in cancer cells. Glutathione enters the cytosol via plasma membrane glutathione transporters such as SLC13A3 or is synthesized through the glutathione biosynthesis pathway. Glutamine-derived glutamate is the major source of glutathione biosynthesis. Most cysteine is taken up by the circulation, and in its oxidized dimer form, cystine is taken up via SLC7A11. Additionally, cysteine can be synthesized from serine and methionine via the transsulfuration pathway. Reduced glutathione can convert hydrogen peroxide and lipid peroxide to water and alcohol, respectively, protecting cells from oxidative damage. OXPHOS: oxidative phosphorylation; ROS: reactive oxygen species; GS: glutathione synthetase; GCL: glutamate–cysteine ligase; GPX: glutathione peroxidases; GR: glutathione reductase; GGT: gamma-glutamyl transferases; LOOH: lipid hydroperoxide; LOH: lipid alcohol; γ-Glu-Cys: gamma-glutamylcysteine.

Consisting of the tripeptide γ-l-glutamyl-l-cysteinyl-glycine, glutathione is the most abundant antioxidant synthesized in cells. Glutathione scavenges free radicals and detoxifies xenobiotics in cells, maintaining cellular redox homeostasis [96]. In cancer cells, increased glutathione levels contribute to tumor growth and chemoresistance [97] (Figure 8).

Increased glutathione levels have been reported in cisplatin resistance in ovarian tumor cell lines [98]. Indeed, in breast cancer cells, overexpression of the apoptotic inhibitor Bcl-2 in MCF-7 cells increased cellular glutathione levels and resistance to cell death in response to cisplatin treatment. Treatment with a glutathione synthesis inhibitor, buthionine sulfoximine (BSO), decreased glutathione levels and abolished Bcl-2-mediated cisplatin resistance, indicating that Bcl-2-mediated cisplatin resistance in cancer cells is dependent on increased glutathione production [99]. In mammary epithelial cells, oncogenic PI3K/AKT stimulation induces glutathione biosynthesis via NRF2-mediated upregulation of glutathione biosynthetic genes. Suppression of glutathione biosynthesis using BSO synergizes with cisplatin, leading to selective tumor regression in PI3K pathway mutant breast cancer cells in vitro and in vivo [100]. Moreover, in ovarian cancer cells, fibroblasts reduce the accumulation of cisplatin in cancer cells, resulting in cisplatin resistance. Mechanistically, fibroblasts provide glutathione and cysteine to cancer cells, overcoming cisplatin-induced DNA damage. Interestingly, CD8-positive effector T cells abolish fibroblast-induced cisplatin resistance via interferon-γ (IFNγ), diminishing fibroblast-derived glutathione and cysteine by upregulating the glutathione catabolic enzyme gamma–glutamyl–transferase (GGT) and through transcriptional repression of SLC7A11 in fibroblasts [101].

However, contradictory results also claim that increased cellular glutathione levels sensitize cancer cells to cisplatin-induced cell death. In epidermal growth factor receptor (EGFR) T790M mutant lung cancer cells, which exhibit resistance to the EGFR inhibitor erlotinib, significantly reduced cellular glutathione levels are observed compared to erlotinib-sensitive cells. Interestingly, increasing glutathione levels in erlotinib-resistant cells using an inhibitor of GST, a glutathione catabolic enzyme, resensitizes resistant cells to erlotinib in vitro and in vivo [102]. Similarly, increasing glutathione by overexpressing GCLC induces hypersensitivity instead of resistance to the cisplatin toxicity via upregulation of the human copper transporter (hCTR1), which also transports cisplatin [103].

### 11.2. Cystine Transportation

The catalytic subunits SLC7A11 (xCT) and SLC3A2 (4F2hc) encode heterodimeric amino acid transport systems that mediate cystine–glutamate exchange and control cellular glutathione levels. Various stress conditions induce SLC7A11 expression via NRF2 and ATF4 to enable adaptation to cellular metabolism. Accordingly, many studies have indicated an association between the expression of SLC7A11 and drug resistance [104] (Figure 8).

Overexpression of SLC7A11 decreased sensitivity to temozolomide with an increased CSC-like phenotype in brain tumor cells [105,106]. Conversely, downregulation of SLC7A11 impaired tumor sphere formation and sensitized CSCs to doxorubicin treatment [107]. In gastric cancer cells, SLC7A11, together with the cellular integrated stress response (ISR) pathway, is responsible for cisplatin resistance. Salubrinal, which activates the ISR pathway, increases intracellular glutathione and reduces cisplatin-induced lipid peroxidation. This cisplatin resistance is diminished by suppression of SLC7A11 and glutathione synthesis [108].

However, systemic inhibition of SLC7A11 may undermine the anticancer immune response, as SLC7A11 is also implicated in supporting T-cell proliferation [109]. Indeed, in culture, T-cell proliferation is strongly dependent on SLC7A11 expression, but SLC7A11 is dispensable for T-cell proliferation and memory immune responses to the tumor in vivo [110]. Thus, combination treatment of SLC7A11 inhibition or cysteine depletion with anticancer immunotherapy using anti-CTLA4 or anti-PD-L1 antibodies dramatically increases the efficacy of anticancer therapy [110,111].

CD44 has been considered a CSC marker in several tumor types [112]. Interestingly, it has been reported that variant isoforms of CD44 containing v8-v10 (CD44v8-10) regulate redox status in cancer cells by stabilizing SLC7A11 and promoting tumor growth, metastasis, and cisplatin resistance [113,114,115,116].

## 12. Amino Acid Metabolism in the Context of the Anticancer Immune Response

### 12.1. Glutamine Plasticity in Immune Cell Metabolism

**Figure 9:**
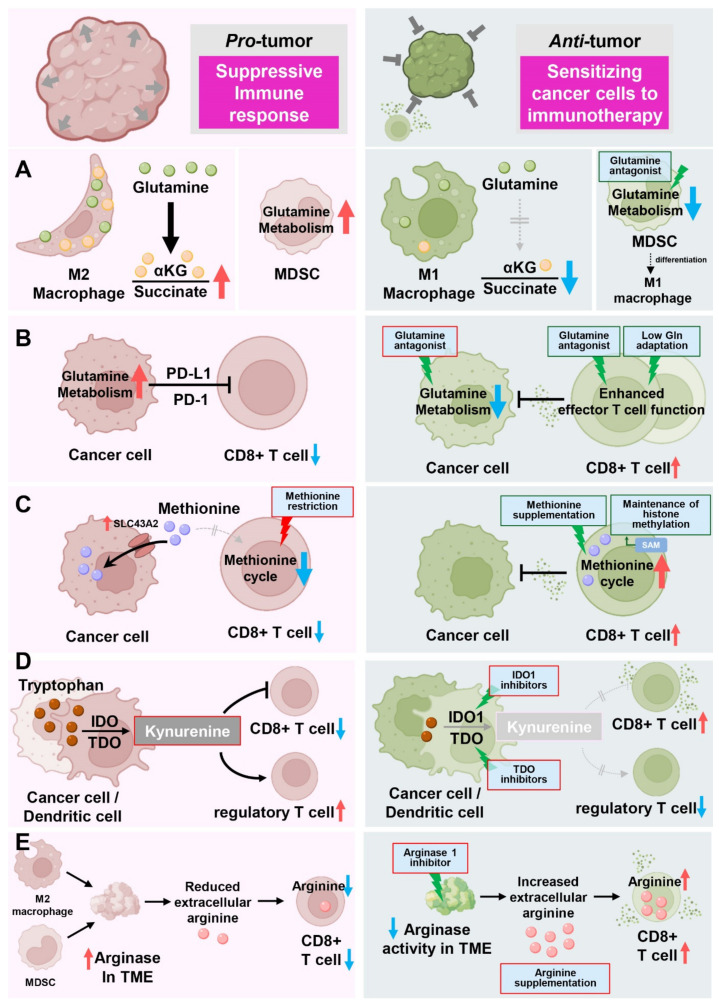
Role of amino acids in the immune response toward cancer cells. (**A**) Glutamine metabolism controls macrophage activation via α-ketoglutarate production, supporting glutamine-induced oxygen consumption and oxidative phosphorylation in protumorigenic M2 macrophages. In MDSCs, inhibition of glutamine usage with JHU083 suppresses the infiltration of MDSCs and induces the differentiation of MDSCs from a suppressive to proinflammatory phenotype, resulting in reduced tumor growth and metastasis. (**B**) Glutamine antagonism using JHU083 conditions CD8^+^ T cells toward an activated and long-lived phenotype, enhancing the anticancer immune response while suppressing glutamine metabolism in cancer cells, resulting in reduced tumor growth. Moreover, glutamine-deprived culture of CD8^+^ T cells leads to reduced tumor growth. (**C**) Cancer cells outcompete T cells for methionine through the methionine transporter SLC43A2 to interfere with T-cell function, reducing H3K79me2 levels. Cancer cells outcompete T cells for methionine through the methionine transporter SLC43A2, which interferes with T-cell function, decreasing H3K79me2 levels. Maintenance of intracellular methionine levels is important for the immune response in T cells. Thus, dietary methionine restriction might be harmful to T cells’ ability to perform anticancer immunity. (**D**) Activity of IDO1 and TDO1 in cancer cells and dendritic cells suppress T-cell function by generating tryptophan-derived kynurenine. Increased levels of kynurenine in tumors promote the differentiation of regulatory T cells via activation of aryl hydrocarbon receptor (AHR) and suppressing effector T-cell functions. IDO1 and TDO1 inhibitors, extensively reviewed elsewhere [125,126], have been intensively investigated for their use in anticancer immunotherapy. (**E**) Arginases secreted from MDSCs or M2-like macrophages in the TME reduce extracellular arginine and suppress T-cell function. Treatment with an arginase inhibitor, genetic ablation of ARG1 in myeloid cells, and arginine supplementation enhance extracellular arginine level and bolster T-cell response.

Exogenous glutamine concomitantly induces drug resistance in cancer cells and generates an immunosuppressive TME [117]. Distinct M2-polarized macrophages in the TME promote tumor progression, including angiogenesis, matrix remodeling, and suppression of the anticancer immune response [118]. Indeed, the production of α-ketoglutarate via glutaminolysis is crucial for the activation of protumorigenic M2 macrophages [119]. This M2 macrophage-inducing mechanism is regulated by a high α-ketoglutarate/succinate ratio, whereas a low ratio strengthens proinflammatory M1 macrophages (Figure 9A).

Myeloid cells comprise a major component of the TME, and myeloid-derived suppressor cells (MDSCs) play important roles in creating an immunosuppressive environment [120]. Impairing glutamine metabolism in MDSCs using a glutamine metabolism–inhibiting prodrug of DON (JHU083) leads to activation-induced cell death and conversion of MDSCs into inflammatory macrophages [121]. Moreover, blocking glutamine metabolism in cancer cells affects the recruitment of MDSCs and increases inflammatory tumor-associated macrophages, rendering checkpoint blockade-resistant tumors susceptible to immunotherapy [121] (Figure 9A).

This blockade of glutamine metabolism is further highlighted by the metabolic plasticity of tumor-infiltrating CD8^+^ effector T cells that, unlike cancer cells, are able to detour glutamine antagonism by inducing metabolic reprogramming toward oxidative phosphorylation [122]. This metabolic flexibility enables CD8^+^ effector T cells to increase their survival and memory functions and to enhance the anticancer immune response [122] (Figure 9B).

The anticancer activity of CD8^+^ effector T cells is often restricted by low nutrient availability in the TME [123]. This hostile metabolic status is highly associated with T-cell exhaustion and deficiency of memory T-cell formation, which are obstacles to successful anticancer adoptive immunotherapy [117]. By adapting chimeric antigen receptor T (CAR-T) cells to conditions containing low glutamine concentrations, it is possible to increase the number of tumor-infiltrating CD8^+^ T cells, leading to normal effector functions of tumor-specific CD8^+^ T cells upon stimulation and promoting memory T-cell differentiation [124] (Figure 9B).

### 12.2. Methionine Dependency in T-Cell Metabolism

Methionine is crucial for T-cell differentiation and activation [127,128]. Upon T-cell activation, methionine metabolism is induced, and exogenous methionine supplies the methyl donor moiety of SAM and maintains H3K4me3 histone methylation in T cells. In this setting, methionine restriction limits the expansion of inflammatory Th17 cells and preserves T-cell-mediated inflammation [128]. Since maintaining the activity of effector T cells present in the TME during cancer treatment is important in anticancer immunotherapy, competition for methionine between cancer cells and immune cells may be a determinant for the prognosis of therapy. Indeed, cancer cells overexpressing the methionine transporter SLC43A2 outcompete T cells for methionine in the TME [129]. This metabolic competition decreases methionine metabolism-related metabolites, including SAM of CD8^+^ T cells, and diminishes H3K79me2, resulting in weakened STAT5-mediated anticancer immunity of effector T cells. Interestingly, methionine supplementation in tumors restored T-cell immunity and reduced tumor sizes [129] (Figure 9C).

These findings indicate a potential side effect in which dietary methionine restriction may weaken the normal anticancer immune response during immunotherapy. Indeed, a recent study showed the opposite results for dietary methionine restriction on tumor progression and therapeutic response in immunodeficient and immunocompetent mice [130]. In contrast to a previous study in which dietary methionine restriction suppressed tumor growth and sensitized tumor cells to chemotherapy [41], dietary methionine restriction enhanced tumor progression and repressed T-cell activation in an immunocompetent mouse colon cancer model. Dietary methionine restriction affects the gut microbiota and reduces fecal hydrogen sulfide (H_2_S), which promotes anticancer immunity through unknown mechanisms [130] (Figure 9C).

### 12.3. Suppressive Effect of Tryptophan-Derived Kynurenine

Tryptophan, an essential amino acid, has been intensively investigated as an immune modulator molecule that affects not only cancer cells but also immune cells. The catabolic process of tryptophan generates several important metabolites, including kynurenine, and rate-limiting step enzymes in the kynurenine pathway, such as indoleamine-2,3-dioxygenase 1 (IDO1) and tryptophan-2,3-dioxygenase (TDO), play critical metabolic roles in the function and survival of immune cells (Figure 9D).

An increased ratio of kynurenine to tryptophan is correlated with a PD-1 blockade-resistance mechanism that is associated with worse overall survival [131,132]. Additionally, an elevated kynurenine-to-tryptophan ratio and kynurenine-mediated immunosuppression have been reported in several tumor types [133,134].

In melanoma, cancer cells release Wnt5a to induce IDO1 activity in dendritic cells, subsequently reducing the efficacy of PD-1 blockade therapy. Furthermore, enhanced IDO1 activity in dendritic cells creates an immunosuppressive TME by exhausting tryptophan supplies that are essential for T-cell activation [135]. Accumulation of kynurenine derived from cancer cells induces regulatory T-cell differentiation and suppresses effector T-cell functions [125,136]. Interestingly, blocking glutamine metabolism also suppressed IDO1 expression in both cancer cells and myeloid-derived cells, leading to a marked decrease in kynurenine levels in a mouse melanoma model [121]. Similarly, tryptophan-derived 3-hydroxyanthranilate is immunosuppressive, directly suppressing effector T-cell activation and promoting regulatory T-cell differentiation [137].

### 12.4. Immunological Function of Arginine in T-Cell Metabolism

Arginine, a conditionally essential amino acid, is involved in diverse biological functions, such as cancer cell growth and survival and immune cell function. Therefore, modulation of arginine availability is becoming highlighted as a promising therapeutic strategy for metabolism-based cancer treatments.

Arginine metabolism is closely linked to T-cell fate and function. Increased arginine levels induced a shift from glycolysis to oxidative phosphorylation in activated T cells and promoted the generation of central memory-like T cells with enhanced survival capacity and anticancer activity [138]. Consistently, in an immunocompetent osteosarcoma mouse model, combination therapy with arginine and anti-PD-L1 antibody therapy bolstered the anticancer immune response [139]. Due to this T-cell dependency on arginine, arginases produced by myeloid cells or macrophages in the TME and consequently depleted extracellular arginine suppress effector T-cell function [140,141]. Thus, inhibiting arginase activity in the TME could be therapeutically exploited to favor the anticancer function of T cells. Indeed, treatment with an arginase inhibitor or genetic ablation of ARG1 in myeloid cells resulted in decreased tumor growth [142,143,144,145] (Figure 9E).

The low arginine availability in the TME also impairs CAR-T-cell proliferation, undermining their efficacy against hematological and solid tumors [146]. The extracellular arginine dependency of T cells partially originates from the low expression of arginine synthesis enzymes in T cells, and exogenous expression of these enzymes might recover effector T-cell function in the TME. Indeed, T cells overexpressing argininosuccinate synthase (ASS) or ornithine transcarbamylase (OTC) display increased CAR-T-cell proliferation without loss of CAR cytotoxicity or T-cell exhaustion in vitro and in vivo [147].

## 13. Outlook

Drug resistance has been the limiting factor for achieving complete cures in cancer patients. The emergence of resistant cancer cells originating from drug-induced selective pressures shows specific resistant metabolic features, including enhanced amino acid metabolism. In this review, we described the role of amino acids in conferring resistance to current chemotherapies, enzyme therapies, and immunotherapies, focusing on the characteristic resistance mechanism of each amino acid.

Recent studies have indicated that targeting amino acid metabolism in cancer cells or immune cells and modification of amino acid composition in the diet can control the efficacy of anticancer treatments. The most common approach targeting amino acid metabolism is the pharmacological suppression of metabolic enzymes that are increased in drug-resistant cancer cells [5,6]. Moreover, the use of modified dietary interventions together with conventional cancer therapy is an approach receiving growing attention owing to its limited toxicity [9,148]. Alteration of the environmental amino acid levels around tumors considerably impacts not only the metabolism of cancer cells but also surrounding cells, including immune and stromal cells, resulting in altered drug sensitivity. However, before clinical use of amino acid supplementation or depletion, the metabolic traits of specific cancer types and their surrounding environment must be characterized to determine the correct amino acid target. Since there is still no consensus or standard guidelines of amino acid sensitizers for improving therapeutic outcomes in cancer patients, additional preclinical and clinical research work focusing on understanding whether and how amino acid modulations suppress cancer in vivo is needed.

In conclusion, knowledge regarding the essential roles of amino acid metabolism in driving drug resistance in cancers has uncovered potential therapeutic approaches for overcoming drug resistance. We hope that more investigations will be performed on the modulation of amino acid metabolism to help reduce the necessity of conventional chemotherapy and related toxicity. Further work in this direction could lead to the design of personalized amino acid modulation that results in many advances in cancer treatment.

## Abbreviations

3-PG	3-phosphoglycerate
5-FU	5-fluorouracil
CH2-THF	5,10-methylene-tetrahydrofolate
ALL	acute lymphoblastic leukemia
AML	acute myeloid leukemia
ASS	argininosuccinate synthase
BCAT1	BCAA transaminase 1
BMSCs	bone marrow stromal cells
BCAAs	branched-chain amino acids
BSO	buthionine sulfoximine
CSCs	cancer stem cells
CAR-T	chimeric antigen receptor T cell
CRC	colorectal cancer
DHFR	dihydrofolate reductase
EGLN1	Egl nine homolog 1
EGFR	epidermal growth factor receptor
EMT	epithelial–mesenchymal transition
ESCC	esophageal squamous cell carcinoma
ER	estrogen receptor
FTCD	formimidoyltransferase cyclodeaminase
GGT	gamma–glutamyl–transferase
GCLC	glutamate–cysteine ligase catalytic subunit
GLUD	glutamate dehydrogenase
GLS1	glutaminase 1
HNSCC	head and neck squamous cell carcinoma
HCC	hepatocellular carcinoma
HAL	histidine ammonia lyase
HER2	human epidermal growth factor receptor 2
IDO1	indoleamine-2,3-dioxygenase 1
iCT	induction chemotherapy
iPS	induced pluripotent stem
ISR	integrated stress response
IFNγ	interferon-γ
ASNase	L-asparaginase
mTORC1	mechanistic target of rapamycin complex 1
MSCs	mesenchymal cells
MAPK	mitogen-activated protein kinase
MDSCs	myeloid-derived suppressor cells
NADPH	nicotinamide adenine dinucleotide phosphate
NSCLC	non-small-cell lung cancer
OTC	ornithine transcarbamylase
PHGDH	phosphoglycerate dehydrogenase
PSAT1	phosphoserine aminotransferase 1
PSPH	phosphoserine phosphatase
ROS	reactive oxygen species
SAH	S-adenosyl homocysteine
SAM	S-adenosyl methionine
SHMT2	serine hydroxymethyltransferase 2
TCA	tricarboxylic acid
TET	ten–eleven translocation
THF	tetrahydrofolate
TNBC	triple-negative breast cancer
TDO	tryptophan-2,3-dioxygenase
TME	tumor microenvironment
TKI	tyrosine kinase inhibitor
α-KG	α-ketoglutarate
